# Inhibitory effect of thiacremonone on MPTP-induced dopaminergic neurodegeneration through inhibition of p38 activation

**DOI:** 10.18632/oncotarget.10504

**Published:** 2016-07-09

**Authors:** Chul Ju Hwang, Hee Pom Lee, Dong-Young Choi, Heon Sang Jeong, Tae Hoon Kim, Tae Hyung Lee, Young Min Kim, Dae Bong Moon, Sung Sik Park, Sun Young Kim, Ki-Wan Oh, Dae Yeon Hwang, Sang-Bae Han, Hwa-Jeong Lee, Jin Tae Hong

**Affiliations:** ^1^ College of Pharmacy and Medical Research Center, Chungbuk National University, Osongsaengmyeong, Osong-eup, Heungduk-gu, Cheongju, Chungbuk, Republic of Korea; ^2^ College of Pharmacy, Yeungnam University, Daehak-Ro, Gyeongsan, Gyeongbuk, Republic of Korea; ^3^ College of Agriculture, Life and Environments Sciences, Chungbuk National University, Osongsaengmyeong, Osong-eup, Heungduk-gu, Cheongju, Chungbuk, Republic of Korea; ^4^ College of Natural Resources & Life Science, Pusan National University, Pusan, Republic of Korea

**Keywords:** garlic, neurodegeneration, P38 MAPK, Parkinson's disease, thiacremonone, Pathology Section

## Abstract

Neuroinflammation is implicated for dopaminergic neurodegeneration. Sulfur compounds extracted from garlic have been shown to have anti-inflammatory properties. Previously, we have investigated that thiacremonone, a sulfur compound isolated from garlic has anti-inflammatory effects on several inflammatory disease models. To investigate the protective effect of thiacremonone against 1-methyl-4-phenyl-1,2,3,6-tetrahydropyridine (MPTP)-induced behavioral impairment and dopaminergic neurodegeneration, 8 week old ICR mice were given thiacremonone (10 mg/kg) in drinking water for 1 month and received intraperitoneal injection of MPTP (15 mg/kg, four times with 2 h interval) during the last 7 days of treatment. Our data showed that thiacremonone decreased MPTP-induced behavioral impairments (Rotarod test, Pole test, and Gait test), dopamine depletion and microglia and astrocytes activations as well as neuroinflammation. Higher activation of p38 was found in the substantia nigra and striatum after MPTP injection, but p38 activation was reduced in thiacremonone treated group. In an in vitro study, thiacremonone (1, 2, and 5 μg/ml) effectively decreased MPP+ (0.5 mM)-induced glial activation, inflammatory mediators generation and dopaminergic neurodegeneration in cultured astrocytes and microglial BV-2 cells. Moreover, treatment of p38 MAPK inhibitor SB203580 (10 μM) further inhibited thiacremonone induced reduction of neurodegeneration and neuroinflammation. These results indicated that the anti-inflammatory compound, thiacremonone, inhibited neuroinflammation and dopaminergic neurodegeneration through inhibition of p38 activation.

## INTRODUCTION

Parkinson's disease (PD) is pathologically characterized by consequent depletion of dopamine in the striatum and dopaminergic neurodegeneration in the substantia nigra [1]. A significant loss of dopaminergic neurons and subsequent striatal dopamine [2] are necessary before symptoms occur, resulting in a clinical diagnosis of the disorder. The main symptoms of PD are brdykinesia (slowness of movement), muscle rigidity and postural instability.

The 1-methyl-4-phenyl-1,2,3,4-tetrahydropyridine (MPTP) animal model has been the most commonly used for inducing PD, because MPTP is a well-known dopaminergic neurotoxin [3] and causes a loss of the nigrostriatal dopaminergic neuron [4] in humans. In addition, MPTP is highly lipophilic, therefore rapidly crosses the blood-brain barrier (BBB) after systemic administration [5]. In the brain, MPTP enters into the astrocytes and metabolizes to MPP+ (active metabolite) by monoamine oxidase B (MAO B). MPP+ causes activation of astrocyte and astrocytic apoptosis [6, 7]. Furthermore, many studies already reported that neuroinflammation can be caused by activation of astrocytes through increased pro-inflammatory cytokines such as TNF-α and IL-1β [8, 9]. In addition, neuroinflammation might contribute to the cascade-dependent neuronal degeneration. A recent study indicated that neuroinflammation is involved in the degeneration of dopaminergic neurons in PD associated animal models [10].

The p38 mitogen-activated protein kinase (MAPK) has been activated in damaged brain tissue and neuronal cell [11, 12]. Moreover, MAO B expression could be regulated by p38 MAP kinase [13]. In activated microglia and astrocytes, inhibition of the p38 MAP kinase pathway prevents MAO B activation [13]. It has been proposed that microglia activation and release of pro-inflammatory cytokines induced by the p38 MAPK pathway, which may contribute to brain damage such as neuronal injury, ischemia and accumulation of oxidants [14]. Thus, it is possible that the inhibition of p38 MAPK pathway could have neuro-protective effects against MPTP induced neurodegeneration.

The transcription factors AP-1 plays a key role in the regulation of several gene expressions including proinflammatory cytokines (TNF-α, IL-1β and IL-6) and inducible enzymes (COX-2 and iNOS) during inflammation, immunity, cell proliferation, stress response, and apoptosis [15–17]. Brain cells like microglia and astrocytes induce and release diverse inflammatory mediators in response to oxidative stress [18–20]. In addition, ROS-induced AP-1 activation can lead to severe inflammatory responses in central nervous systems (CNS) [21]. These studies implicate that expression or activation of AP-1 leads to inflammatory genes expression in brain inflammation and neurodegenerative disorders. Furthermore, AP-1 appears to be influenced by p38 through several different mechanisms. The p38 MAPK indirectly regulates AP-1 activity through regulating ATF-2 and c-fos [22].

Garlic has been used in traditional medicine as a food component to prevent the development of various diseases including neurodegenerative disease [23–29]. These pharmacological effects of garlic can be attributed to the presence of the antioxidant substance, thiacremonone (2,4-dihydroxy-2,5-dimethyl-thiophene-3-one) [30]. Our group reported that this compound has anti-oxidative activities [31] as well as anti-inflammatory and anti-cancer effects *in vitro* and *in vivo* [24, 32–36]. In the present study, we investigated the protective effect of thiacremonone on MPTP-induced neuro degeneration through the down-regulation of p38 pathway.

## RESULTS

### Effect of thiacremonone on behavioral impairments

**Figure 1:**
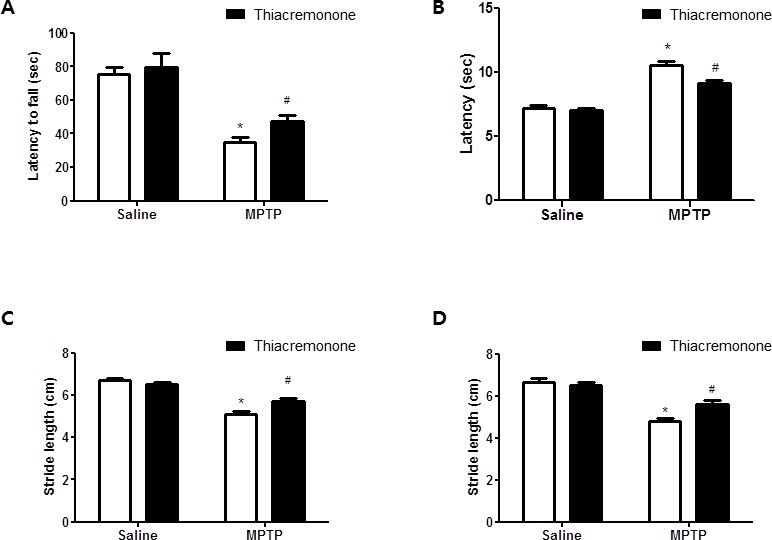
Thiacremonone ameliorates MPTP-induced behavior disorder Performance on the rotarod is impaired in MPTP injected groups. However, impairment is ameliorated in MPTP-injected thiacremonone treated groups **A.** MPTP-induced bradykinesia is ameliorated in MPTP-injected thiacremonone treated groups **B.** Stride length of forelimb **C.** and hindlimb **D.** are more increased by thiacremonone treatment in MPTP injection groups. Each value is presented as mean ± SD from 10 mice. *, *p* < 0.05 Significant difference from saline-injected mice and #, *p* < 0.05 Significant difference between the MPTP-injection groups.

Treatment of thiacremonone did not cause any behavioral difference between saline injection groups. The rotarod test was carried out to assess coordination capability of four groups. MPTP treatment significantly decreased latency to fall from a treadmill in both control and thiacremonoe treated groups. However, the decrement of latency was significantly lower in thiacremonone-treated mice (48.3 ± 4.25 s) compared to MPTP-treated mice (36.2 ± 3.24 s) (Figure 1A). Next, we conducted the pole test and measured the time until the mice took to descend from the top of the pole to the floor. Elongation of the parameter is considered to reflect bradykinesia. Saline injection and thiacremonone itself did not induce the significantly change of behavioral function. In contrast, the time to descend was significantly delayed by MPTP injection, but the delay of time was significantly less in thiacremonone-treated mice (9.7 ± 0.88 s) compared to MPTP-treated mice (10.96 ± 0.98 s) (Figure 1B). When stride length test was performed, results showed that MPTP injection shortened fore limb stride length (Figure 1C) as well as lengths of hind limb (Figure 1D). However, the stride length was less shortened in thiacremonone-treated mice (Fore limb: 5.7 ± 0.09 s, Hind limb: 5.4 ± 0.12 s) compared to MPTP-treated mice (Fore limb: 5.4 ± 0.11 s, Hind limb: 4.8 ± 0.21 s).

### Effect of thiacremonone on the expression of GFAP and iBA1

**Figure 2:**
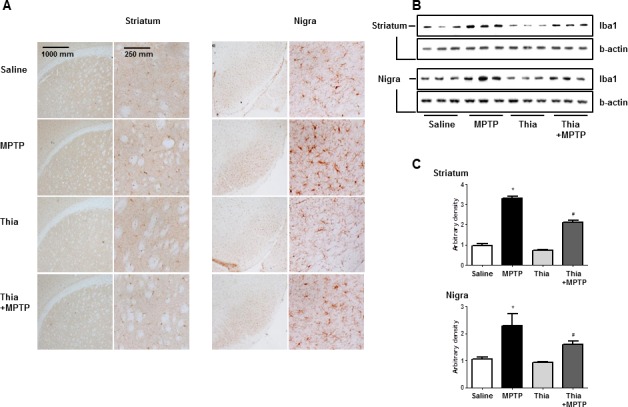
Thiacremonone reduces the expression of iBA1 The effect of thiacremonone on reactive microglia cells were measured by immunohistochemical analysis and Western blotting analysis. The sections of mice brain (striatum and substantia nigra) incubated with anti-iBA1 primary antibody and the biotinylated secondary antibody (*n* = 3). The representive stained tissues were viewed with a microscope (X50 or 200) **A.** Expression of iBA was also examined by specific antibodies in the brain **B.** The graph represents Arbitrary density of each blots **C.** Each value is presented as mean ± SD from 10 mice. *, *p* < 0.05 Significant difference from saline-injected mice and #, *p* < 0.05 Significant difference between the MPTP-injection groups.

**Figure 3:**
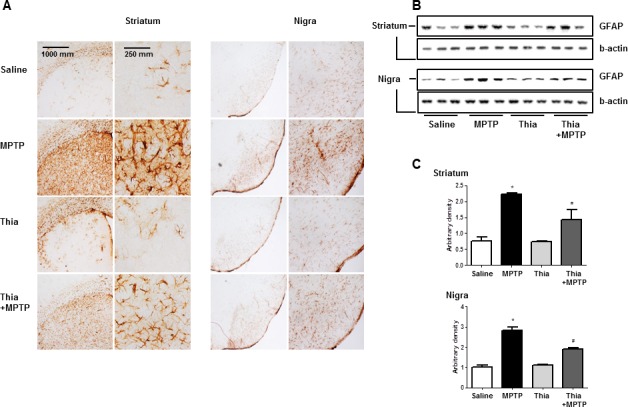
Thiacremonone reduces the expression of GFAP The effect of thiacremonone on reactive astrocytes was measured by immunohistochemical analysis and Western blotting analysis. The sections of mice brain (striatum and substantia nigra) incubated with anti-GFAP primary antibody and the biotinylated secondary antibody (*n* = 3). The represented stained tissues were viewed with a microscope (X50 or 200) **A.** Expression of GFAP was also examined by specific antibodies in the brain **B.** The graph represents Arbitrary density of each blots **C.** Each value is presented as mean ± SD from 10 mice. *, *p* < 0.05 Significant difference from saline-injected mice and #, *p* < 0.05 Significant difference between the MPTP-injection groups.

Neuroinflammation is critical for the development of parkinson disease, and it can be induced by the activation of astrocytes and microglia. To determine whether MPTP injection can induce neuroinflammation as well as activate astrocytes and microglia, Western blot and immunohistochemistry were used to detect the expression of GFAP (a marker of astrocytes activation) and iBA1 (a marker of microglia activation) in mouse brains. Our data indicated that the numbers of reactive cells of immunostaining for GFAP (Figure 2A) and iBA1 (Figure 3A) in striatum and substantia nigra of MPTP-injected thiacremonone-treated mice were significantly lower compared to the numbers in MPTP-treated mice. MPTP-induced proteins expression of GFAP (Figure 2B and 2C) and iBA1 (Figure 3B and 3C) in striatum and substantia nigra was also significantly decreased in thiacremonone-treated mice compared to MPTP-treated mice.

### Effect of thiacremonone on dopaminergic neurodegeneration

**Figure 4:**
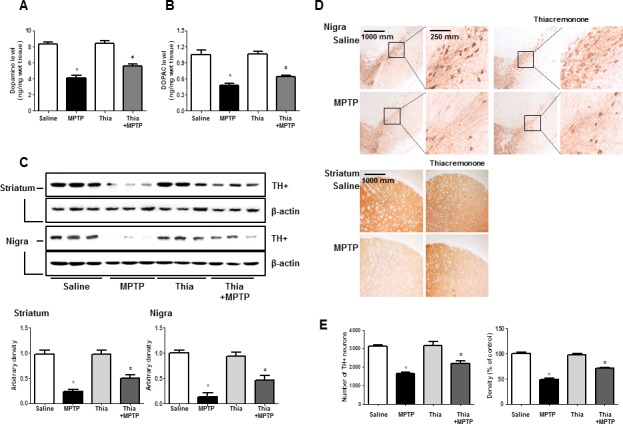
Thiacremonone ameliorates MPTP-induced dopaminergic neurodegeneration Level of dopamine **A.** and DOPAC (which is metabolite of dopamine) **B.** in the 4 groups of mice brain were determined by HPLC. Method was as described detail in materials and methods section. The effect of thiacremonone on TH-positive neurons was measured by western blot and immunohistochemical analysis. Expression of TH was examined by specific antibodies in the brain striatum and substantia nigra **C.** The sections of mice striatum and substantia nigra **D.** incubated with anti-TH+ primary antibody and the biotinylated secondary antibody (*n* = 3). The representive stained tissues were viewed with a microscope (X50 or 200). Represents the number of TH-positive neuronal cells in substantia nigra and TH-positive density in striatum **E.** Total cell number counted in each whole section. Each value is presented as mean ± SD from 10 mice. *, *p* < 0.05 Significant difference from saline-injected mice and #, *p* < 0.05 Significant difference between the MPTP-injection groups.

PD could result in the depletion of dopamine and (or) its metabolites. In addition, activation of astrocytes metabolite MPTP to MPP+ that might deplete dopamine and cause neuronal cell death. To investigate whether thiacremonone-induced inactivation of astrocytes could prevent dopamine depletion and neuronal cell death, we determined the level of dopamine and its metabolites. To quantify the neurochemicals including dopamine and its metabolite DOPAC, HPLC analysis were conducted with striatum tissues. Dopamine concentration in the striatum of MPTP-injected thiacremonone-treated mice was significantly higher than that of MPTP-treated mice (Figure 4A). In addition, treatment of thiacremonone shows lower levels of DOPAC in the striatum after MPTP injection (Figure 4B). We evaluated MPTP-neurotoxicity using Western blot and immunohistochemical stainings for TH. MPTP-induced proteins expression of TH in striatum and substantia nigra was also significantly increased in thiacremonone-treated mice compared to MPTP-treated mice (Figure 4C). There were abundant TH-positive fibers in the striatum and substantia nigra in saline injected groups. When the mice were injected with MPTP, the number of TH-positive neurons was significantly lowered in the substantia nigra as determined by immunostaining and stereological counting. However, population of the dopaminergic neuron after MPTP-intoxication was recovered in thiacremonone-treated mice compared to MPTP-treated mice (Figure 4D). Consistent with this, density of TH-positive fibers in the striatum was higher and number of TH-positive cells in the nigra was higher in thiacremonone-treated mice (Figure 4E).

### Effect of thiacremonone on MAO B expression, activation of MAPK, DNA binding activity of AP-1 and expression of neuroinflammation marker proteins

**Figure 5:**
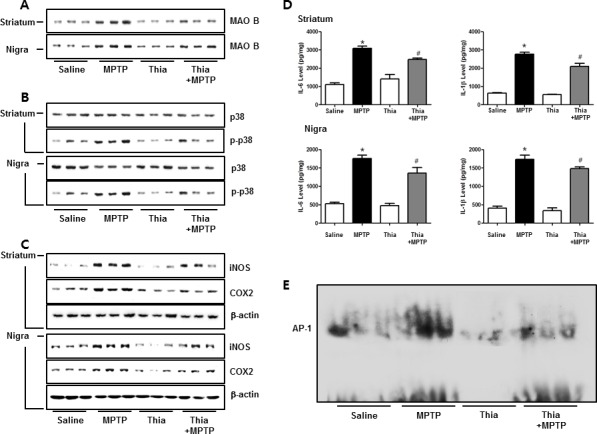
Thiacremonone reduces neuroinfalmmation by p38 pathway The effect of thiacremonone on neuroinflammation, expression of inflammatory proteins was measured by Western blotting analysis. Expression of MAO B was examined by specific antibodies in the striatum and nigra regions of brain **A.** Avtivation of p38 was determined by specific antibodies in the striatum and substantia nigra regions of brain **B.** Expression of iNOS and COX2 were examined by specific antibodies in the striatum and substantia nigra regions of brain **C.** Levels of pro-inflammatory cytokines, IL-6 and IL-1β were measured in substantia nigra regions of brain **D.** The AP-1 DNA binding activity was measured by EMSA **E.** Each value is presented as mean ± SD from 10 mice. *, *p* < 0.05 Significant difference from saline-injected mice and #, *p* < 0.05 Significant difference between the MPTP-injection groups.

MAO B is responsible for the metabolism of MPTP, which generates neurotoxic metabolite MPP+. Thus, we determined MAO B expression by Western blotting. Immunoblot data indicated that MPTP-induced expression of MAO B was much lower in the striatum and nigral region of thiacremonone-treated mice than that of MPTP-treated mice (Figure 5A). It is well known that astrocytes are important cells contributing expression of MAO B, responsible for MPTP metabolism. Evidence suggests that MAO B expression is regulated by p38 MAPK activity [13]. Furthermore, p38 MAPK activation is accompanied by astrogliosis [37]. Thus, we speculated that downregulated expression of MAO B in thiacremonone-treated mice might be associated with inactivation of the p38 MAPK pathway. In support of our assumption, MPTP-induced phosphorylation of p38 MAPK was significantly reduced in thiacremonone-treated mice brain compared to MPTP-treated mice brain (Figure 5B). It is well established that neuroinflammation is critical for dopaminergic neurodegeneration. To determine whether thiacremonone treatment can reduce neuroinflammation, we used the Western blot to detect the expression of COX2 and iNOS in mouse striatum and nigral regions. MPTP-induced expressions of COX2 and iNOS were much lower in the striatum and nigral region of thiacremonone-treated mice brain (Figure 5C). Pro-inflammatory cytokines, IL-1β and IL-6, play important roles in neuroinflammatory disease. MPTP-injection significantly elevated the IL-1β and IL-6 levels in mice striatum and nigra regions. However, MPTP-induced IL-1β and IL-6 release were inhibited in thiacremonone treated mice striatum and nigra (Figure 5D). The activation of AP-1 plays a critical role in neuro-inflammation since it controls several genes involving neuroinflammation. To determine whether treatment of thiacremonone could decrease the activation of AP-1 after MPTP injection, we measured the DNA binding activity of AP-1 by EMSA. MPTP-injection significantly elevated DNA binding activity of AP-1 in mice brain. However, much lower DNA binding activity of AP-1 was observed in the brain of thiacremonone-treated mice compared to MPTP-treated mice brain (Figure 5E).

### Effect of the thiacremonone on MPP+-induced neuroinflammation in BV-2 cells and neuronal cell death in cultured neuronal cells

**Figure 6:**
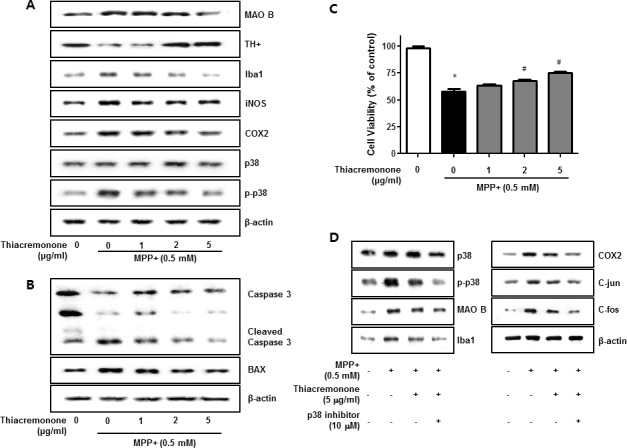
Effect of thiacremonone against dopaminergic neurodegeneration and apoptosis *in vitro* The protection effect of thiacremonone against neurodegeneration, expressions of TH, MAO B and inflammatory marker proteins were measured by Western blotting analysis. Avtivation of p38 was determined by specific antibodies, and expressions of TH, MAO B and inflammatory marker proteins (Iba1, iNOS, COX2) were examined by specific antibodies in BV-2 cells **A.** To evaluate the protection effect of thiacremonone against neuronal cell apoptosis, cell viability and expression of apoptotic proteins was measured by Western blotting analysis. Expression of BAX and caspase 3 was examined by specific antibodies in cultured neuronal cells **B.** The cell viability was masured by MTT assasy **C.** To involvement of p38 pathway in the inhibitory effect of thiacremonone on MPP+-induced neuroinflammation. BV-2 cells were inhibited with specific p-38 inhibitor (10 μM) for 24 h, treated with thiacremonone (5 μg/ml) at 37°C. These effects were measured by western blot analysis. Expressions of MAO B, inflammation marker proteins (Iba1, COX2, and iNOS) and AP-1 subunit (c-jun/c-fos) were examined by specific antibodies **D.** *, *p* < 0.05: Significant difference from the MPP+-treated astrocytes and #, *p* < 0.05: Significant difference between the astrocytes treated with β-estradiol and cotreated with p38-specific inhibitors.

To evaluate effect of thiacremonone on MPP+-induced neurodegeneration and neuroinflammation, we investigated the participation of the p38 pathway in cultured BV-2 cells. Thiacremonone increased TH expression but decreased MAO B expression induced by MPP+ treatment in BV-2 cells (Figure 6A). Moerover, MPP+-induced neuroinflammatory proteins, COX2, iNOS and Iba1 expressions were decreased in BV-2 cells in a dose dependent manner by thiacremonone (Figure 6A). MPP+ could promote the neuronal apoptosis. To further study the preventive effect of thiacremonone in cultured neuronal cell, we determined expression of apoptotic proteins. MPP+-induced expressions of BAX and caspase3 cleavage were decreased by thiacremonone treatment in a dose dependent manner in cultured neuronal cells (Figure 6B). In addition, MPP+-induced reduction of cell viability was recovered by thiacremonone treatment in a concentration dependent manner in cultured neuronal cell (Figure 6C).

### Involvement of the p38 pathway in the inhibitory effect of thiacremonone on MPP+-induced neuroinflammation

In order to further examine the mechanisms regulating neuroinflammation by the p38 pathway, we used specific inhibitors in BV-2 activated by MPP+, and investigated the involvement of the p38 pathway in neuroinflammation. Thiacremonone decreased MAO B as well as pro-inflammatory proteins (COX2, iNOS and Iba1) expression by MPP+ treatment in BV-2 cells. However, the thiacremonone-induced inhibitory effect was amplified by the inhibition of p38 activation in BV-2 cells (Figure 6D). Thiacremonone also decreased the expression of the C-jun and C-fos expression induced by MPP+ treatment in nuclear lysates of BV-2 cells. These inhibitory effects were enhanced by the inhibition of p38 activation in nuclear lysates of BV-2 cells (Figure 6D).

### Binding between Tiacremonone and activated p38

**Figure 7:**
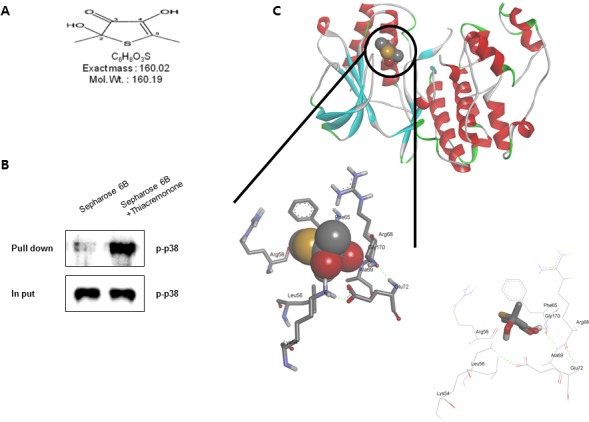
Structure of thiacremonone and activated p38, and binding of thiacremonone to activated p38 Structure of thiacremonone, a sulfurcompound isolated from garlic **A.** Whole cell lysates of BV-2 were incubated with thiacremonone-conjugated Sepharose 6B. After precipitation, the levels of bound activated p38 were monitored by Western blot analysis **B.** Ribbon representation docking model of thiacremonone with activated p38 and Molecular surface representation docking model of thiacremonone with activated p38 **C.**

To clear the p38 involvement in the inhibitory effect of thiacremonone MAO B and neuroinflammatiory protein expression, we determined the interaction between thiacremonone and p38. The interaction was first assessed in a pull-down assay using thiacremonone (Figure 7A)-Sepharose 6B beads, and the binding was detected by immunoblotting with anti-p-p38. The results indicated that thiacremonone bound with cell lysates containing p-p38 protein from BV-2 cells (Figure 7B). To identity the binding site of thiacremonone to activated p38, we performed a computational docking model of thiacremonone with activated p38 under the Schrödinger docking program, Glide. The results from the docking studies suggested that thiacremonone might covalently bind with Gly 170 residue in activated p38 catalytic site (Figure 7C).

## DISCUSSION

Accumulating evidences have suggested that neuroinflammation may contribute to the occurrence and progression of PD [38, 39]. The brains of PD patients appear to show high hallmarks of neuroinflammation, including marked astrogliosis, and elevated release of proinflammatory mediators and cytokines, as well as microglial activation [40]. Indeed, neuroinflammation has been reported to cause dopaminergic cell death in several PD models. LPS-induced (single intranigral injection) PD rat model showed higher rate of death of dopaminergic neurons in the substantia nigra [41]. Chronic release of pro-inflammatory cytokines by activated astrocytes and microglia leads to the exacerbation of DA neuron degeneration. In addition, neuroinflammation is also implicated in the pathogenesis of PD [42]. Recent studies indicate that microglia was activated in various regions of PD brain [43]. Moreover, higher activation of microglia in the nigra and striatum was observed in various types of PD animal models [44–47]. Therefore, many anti-inflammatory agents have been suggested as possible compounds for PD treatment.

Many studies have shown that fresh garlic extracts, garlic oil, garlic-derived chemicals and specific organo sulfur compounds generated by processing garlic could have neuroprotective effects via its anti-neuroinflammatory and anti-oxdative properties [23, 25, 48]. Allicin, isolated from galin oil, showed the strongest inhibition of acetylcholinesterase and butyrylcholinesterase enzymes [49]. Futhermore, allicin attenuates ischemic brain injury *in vivo* and *in vitro* studies [50–52]. S-allyl cysteine (SAC), the most abundant organosulfur compound in garlic extract, activates the antioxidant responses and protects neurons against ischemic injury *in vitro* and *in vivo* [53, 54]. Thiacremonone was isolated as a sulfur compound from a hot water extract of garlic, and found that this compound has an anti-cancer effect on colon cancer and lung cancer [32, 34]. Our present data showed that thiacremonone decreased MPTP-induced behavioral disorder, dopamine depletion and microglia and astrocytes activations as well as neuroinflammation. In an *in vitro* study, thiacremonone effectively decreased MPP+-induced glial activation, inflammatory mediators generation and dopaminergic neurodegeneration. Previously we have previously reported that thiacremonone has an anti-inflammatory effect in several inflammatory disease models such as alzheimer's disease, hepatic failure and arthritis [24, 30, 31, 55]. In the present study, thiacremonone also inhibited dopaminergic cell death via its anti-neuroinflammatory properties in MPTP-injected mice as well as cultured microglia and neuronal cells. These results indicated that like other sulfur compound from garlic, thicremonone has anti-neuroinflammatory effects.

Dopaminergic neurons are located in the substantia nigra. Neurons of the substantia nigra communicate with other neurons by liberating the neurotransmitter DA. In PD patient's brain, neurons of the nigra progressively degenerate, and this loss of neurons in nigra can cause reduced amount of DA available for neurotransmission [56]. Reductions of DA content in brain tissue from patients with PD have been documented in a variety of studies [57–59]. Dopamine synthesis can be promoted by TH through hydroxylation of tyrosine to L-DOPAThese results support that thicremonone could be an effective compound for neurodegenerative disease, and this effect can protect the brain against PD risk.

In recent studies, activation of p38 MAPK was increased in MPTP-induced PD animal models as well as PD patient brain [63, 64]. The activation of the p38 pathway plays an important role in the production of proinflammatory cytokines (IL-1β, TNF-α and IL-6) [65–67] as well as expression of inflammatory proteins such as COX-2 and iNOS [68, 69]. Thus, p38 MAPK pathway is significant for PD-induced neuroinflammation. Moreover, p38 pathway can be also involved in the MAO B expression through astrogliosis. In recent studies, MAO B expression and astrogliosis were attenuated by inhibition or knockout of p38 MAPK [13, 37, 70]. In addition, up-regulation of AP-1 activation can lead to the development of inflammatory disease through increased cytokines release [71]. Recently, it has been shown that p38 MAPK pathways can induce AP-1 activity in PD patient brain and animal model [72–74]. In the present study, we observed much decreased MPTP-induced neuroinflammatory protein expression (iNOS and COX2), astrocyte and microglia activation as well as MAO B expression and cytokine releases accompanied with activation of p38 pathway in the brain of thiacremonone treated mice compared to vehicle treated mice. In addition, thiacremonone inhibited AP-1 binding activity and c-fos/c-jun expression. Furthermore, these inhibitory effects were amplified by treatment of p38 specific inhibitor. Moreover, we observed that thiacremonone can direct-binding with phosphorylated p38 by 3D docking analysis, and we confirmed this using *in vitro* pull-down assay. These evidences suggest that thiacremonone can inhibit neuroinflammation through inactivation of astrocyte via blocking of p38 MAPK pathway. In our previous study, we did not detect any side effects of thiacremonone (loss of weight or any observed toxic sign) on the effective dose of thiacremonone (10 mg/kg) during treatment for 20 days [24]. This evidence suggests that thiacremonone may be potentially beneficial for the prevention of inflammatory diseases with comparatively low toxic effects.

In our recent studies, the anti-inflammatory effects of thiacremonone in several disease models were reported [24, 30, 31, 55]. In APAP induced-hepatic disease model, thiacremonone has a curative effect through Inhibition of Proinflammatory Cytokines Production [55]. Also, thiacremonone showed the anti-inflammatory effect on TPA-induced ear edema and carrageenan-induced arthritis [24]. In case of neuropathies, thiacremonone has an anti-inflammatory effect in several of our models. LPS-induced neuro-inflammation was significantly reduced by treatment of thiacremonone [30]. Also, thiacremonone significantly attenuated cognitive impairments through stimulating anti-oxidant system in a double-mutant transgenic AD mice model [31]. The key mechanisms of these therapeutic effects of thiacremonone were derived by regulation of NF-ĸB. NF-ĸB is a transcription factor which can promote various inflammation-related genes transcription [75]. However, transcriptional activation of NF-ĸB can be controlled by p38 MAPK [76]. In the present study, thiacremonone can block p38 MAPK pathway through direct binding with phosphorylated p38 protein. This double-inhibitory effect of NF-ĸB activation through both directly and p38 pathway mediated can amplify the anti-inflammatory effect of thiacremonone. Thus, our present study further suggests that thiacremonone could be a possible compound for the pharmacotherapy for neuroinflammatory injury in PD.

## MATERIALS AND METHODS

### Extraction and characterization of thiacremonone

The structure of a sulfur compound isolated from garlic (named thiacremonone) as shown previously (Figure 1A). Preparing method of thiacremonone was done as described previously [35]. This fraction containing thiacremonone was purified by preparative RP-HPLC on a Younglin SP930D Instrument [30]. Thiacremonone was resolved in distilled water.

### Animals and MPTP treatment

Mice were purchased from DBL Co., Ltd (Eumseong-gun, Korea). All of the experimental procedures were approved by the Animal Care and Use Committee (IACUC) of Chungbuk National University (approval number: CBNUA-144-1001-01). Four experimental groups of mice (*n* = 10) were studied: the vehicle (Sterile water) treated group1/2 (*n* = 20) and the thiacremonone treated group1/2 (*n* = 20). All were male, weighing 25-30 g (10 week-old) at the time of exposure. The mice were given vehicle or thiacremonone (10 mg/kg) in drinking water for 1 month. All mice were housed in a room that was automatically maintained at 21-25°C and at relative humidity (45-65%) with a controlled light-dark cycle. Mice were administered intraperitoneal injections of MPTP (15 mg/kg, Sigma-Aldrich, St Louis, MO) or saline four times daily at 2 h intervals for 7 days. Mice were sacrificed 9 days after (In order to access the behavioral test) the last injections for experimental analyses.

### BV-2 cell culture

BV-2 cells were obtained from the American Type Culture Collection (Rockville, MD). These cells were maintained at subconfluence in a 95% air, 5% CO2 humidified atmosphere at 37°C. The medium used for routine subcultivation was Dulbecco's Modified Eagle's Medium (DMEM, Invitrogen, Carlsbad, CA), supplemented with 10% fetal bovine serum (FBS), penicillin (100 units/ml). Cells were counted with a hemocytometer and the number of viable cells was determined through trypan blue dye exclusion.

### Neuronal cell culture

The Sprague-Dawley rats were maintained in accordance with the policy of the National Institute of Toxicological research, which is in accord with the Korea Food and Drug Administration's guideline for the care and use of laboratory animals. Sprague-Dawley rats weighing 200-300 g were housed under 12 h rlight/dark cycles, at 23°C and 60 ± 5% humidity. All animals had free access to food (Samyang Foods, Seoul, Korea) and water. Cerebral cortical cells were isolated from neonatal rat brain (Day 1) in PBS (0.1 mol). Briefly, cerebral cortices were removed and incubated for 15 min in Ca2+- and Mg2+-free Hanks' balanced saline solution (Life Technologies) containing 0.2% trypsin. Cells were dissociated by trituration and plated into polyethyleneimine-coated plastic or glass-bottomed culture dishes containing minimum essential medium with Earle's salts supplemented with 10% heat-inactivated fetal bovine serum, 2 mM l-glutamine, 1 mM pyruvate, 20 mM KCl, 10 mM sodium bicarbonate, and 1 mM Hepes (pH 7.2). Following cell attachment (3-6 h after plating), the culture medium was replaced with neurobasal medium containing B27 supplements (Life Technologies). The cells were cultured in neuronal cell culture medium for 3 days, and then further cultured in neuronal cell culture medium (NCM) with or without 20% astrocyte culture media (ACM). Experiments were performed with 4 to 6-day-old cultures; greater than 90% of the cells in these cultures were neurons, and the remainder were astrocytes, as judged by cell morphology and by immunostaining with antibodies against neurofilaments and glial fibrillary acidic protein.

### Cell viability assay

Cell viability assay was evaluated using a WST-8 assay (Dojindo Laboratories, Tokyo, Japan). WST-8 [2-(2-methoxy-4-nitrophenyl)-3(4-nitro-phenyl)-5-(2,4-disulfo-phenyl)-2H-tetrazolium,monosodium salt] is reduced by de-hydro-genases in cells to give a yellow-colored product (formazan), which is soluble in the culture medium. The amount of the formazan dye generated by the activity of dehydro-genases in cells is directly proportional to the number of living cells. In brief, 1 × 104 cells per well were plated into 96-well plates, incubated at 37°C for 24 h, and given a fresh change of medium. Cells were then incubated with or without LPS (1 μg/ml) in the absence or presence of various concentrations of ent-Sauchinone at 37°C for an additional 24 h. At that point, 10 μl of the WST-8 solution was added to the wells and incubation was continued for another 1 h. The resulting color was assayed at 450 nm using a microplate absorbance reader (SunriseTM, TECAN, Switzerland).

### Behavioral tests

We performed behavioral tests 2 days after the last MPTP injection to examine whether there is a difference in the neurotoxicant-caused behavioral deficit between the thiacremonone treated group and control group. Rotarod, pole, and gait tests were conducted as the behavioral tests.

### Rotarod test

The rotarod test was done as described previously [77]. Mice were trained for two consecutive days before MPTP injections in acceleration mode (2-20 rpm) over 5 min. The training was repeated with a fixed speed (16 rpm) until the mice were able to stay on the rod for at least 300 s.

### Pole test

The pole test was done as described previously [77]. The test trials were performed three times per animal, and average values from three examinations were used for each animal.

### Gait test

The gait test was done as described previously [77]. Mice were subject to two training trials to be acclimatized in the environment. A single test trial was performed, and stride length was measured as the distance between successive paw prints. Data were presented as the average of five strides for each animal.

### Collection and preservation of brain tissues

After the behavior tests, mice were anaesthetized with CO2 gas and then perfused with phosphate-buffered saline (PBS). The brains were immediately removed from the skull, and the tissues were stored at −80°C until biochemical analysis.

### Molecular Modeling

The crystal structure of p38 was used for the docking study. The thiacremonone was built using a Maestro build panel. The compound was minimized using the Impact module of Maestro in the Schrödinger Suite Program. The starting coordinate of the p38 was further modified for binding model prediction. The protein structure was minimized using the Protein Preparation Wizard by applying an OPLS force field. For the grid generation, the binding site was defined as the centroid of the Gly 170 in the catalytic site of p38. Ligand docking into the catalytic site of p38 was carried out using the Schrödinger docking program, Glide. The minimized conformation of thiacremonone was docked into the prepared receptor grid. The best-docked poses were selected as the initial covalent model of Gly 170 in the catalytic site of p38 with thiacremonone. The covalent complexes were further minimized using the steepest descent algorithm. Molecular graphics for the covalent binding model of the thiacremonone was generated using a PyMol package (http://www.pymol.org).

### Pull down assay

Thiacremonone was conjugated with cyanogen bromide (CNBr)-activated Sepharose 6B (Sigma-Aldrich, St. Louis, MO). Briefly, thiacremonone (1 mg) was dissolved in 1 ml of coupling buffer (0.1 M NaHCO3 and 0.5 M NaCl, pH 6.0). The CNBractivated Sepharose 6B was swelled and washed in 1 mM HCl through a sintered glass filter, then washed with the coupling buffer. CNBr-activated Sepharose 6B beads were added to the thiacremonone-containing coupling buffer and incubated at 4°C for 24 hr. The thiacremonone-conjugated Sepharose 6B was washed with three cycles of alternating pH wash buffers (buffer 1, 0.1 M acetate and 0.5 M NaCl, pH 4.0; buffer 2, 0.1 M Tris-HCl and 0.5 M NaCl, pH 8.0). Thiacremonone-conjugated beads were then equilibrated with a binding buffer (0.05 M Tris-HCl and 0.15 M NaCl, pH 7.5). The control unconjugated CNBr-activated Sepharose 6B beads were prepared as described above in the absence of thiacremonone. The cell lysate was mixed with thiacremonone-conjugated Sepharose 6B or Sepharose 6B at 4uC for 24 hr. The beads were then washed three times with TBST. The bound proteins were eluted with SDS loading buffer. The proteins were then resolved by SDS-PAGE followed by immunoblotting with antibodies against p-p38 (1:1,000 dilution, Abcam, plc. Cambridge, UK).

### HPLC analysis of dopamine and its metabolite

Dopamine and metabolites in the striatum were measured by HPLC. Briefly, tissues were sonicated in chilled 0.1 M perchloric acid containing dihydroxybenzylamine as an internal standard. After centrifugation (15,000×g, 30min, 4°C), supernatant was diluted with mobile phase (75 mM of NaH2PO4, 1.7 mM Octane sulfonic acid, 10% methanol, pH 3.0), and 10 μl of sample was isocratically eluted through a 80×4.6 mm C18 column (Waters Associates, Milford, MA) with flow rate of 1.5 ml/min. Neurochemicals including dopamine, 3,4-dihydroxyphenylacetic acid (DOPAC) were detected by a two-channel electrochemical detector (Waters Associates) at a potential of 1.5 mV. Concentrations were normalized by wet tissue weight.

### Measurement of cytokines level

Lysates of whole brain tissue were obtained through protein extraction buffer containing protease inhibitor. Dopamine levels were also determined using each specific ELISA Kit (Wuhan Huamei Biotech Co., Ltd., Hubei, China). In brief, 100 μl of sample was added into the precoated plate and incubated for overnight at 4°C. After washing each well of the precoated plate with washing buffer, 100 μl of labeled antibody solution was added, and the mixture was incubated for 1 h at 4°C in the dark. After washing, chromogen was added, and the mixture was incubated for 30 min at room temperature in the dark. Finally, the resulting color was assayed at 450 nm using a microplate absorbance reader (SunriseTM, TECAN, Switzerland) after adding stop solution.

### Immunohistochemical staining

The immunohistochemical staining was done as described previously [77]. The sections were incubated for 2 h at room temperature with a rabbit/mouse polyclonal antibody against glial fibrillary acidic protein (GFAP), iba1 (1:300; Abcam, Inc., Cambridge, MA), a goat polyclonal antibody against MAO B (1:300; Santa Cruz Biotechnologies, Inc., Santa Cruz, CA). After incubation with the primary antibodies, sections were washed in PBS before being incubated for 1 h at room temperature in the presence of biotinylated anti-goat/mouse/rabbit IgG secondary antibodies (1:1000; Vector Laboratories, Burlingame, CA). Eight mice per group underwent immunohistochemical staining.

### Analysis of the number of TH-positive neurons and density of TH-positive fibers

The total number of TH-positive cells was counted in sections using the optical fractionator method for unbiased cell counting as described previously with slight modification [77]. Briefly, every sixth section throughout the entire extent of the substantia nigra was picked, and immunostaining for TH was performed. The number of TH-positive neurons was counted by using a computer-assisted image analysis system consisting of a Zeiss Axioskop2 Plus photomicroscope equipped with an MS-2000 (Applied Scientific Instrumentation, Eugene, OR) computer-controlled motorized stage, a Sony DXC-390 video camera, a DELL GX260 workstation, and the Optical Fractionator Project module of the BIOQUANT Stereology Toolkit Plug-in for BIOQUANT Nova Prime software (BIOQUANT Image Analysis Corporation, Nashville, TN). The Substantia nigral region was observed at a low magnification (10× objective) and was outlined by using a set of anatomical landmarks. The cell number was counted at a high magnification (40× objective). After counting was finished, the total number of neurons was automatically calculated by the software. For determining striatal TH-positive fiber density, six striatum-containing sections covering the entire head and tail of the striatum from each animal were chosen. TH-positive fiber density was measured by using Image Analysis software (Image Lab). Each value was corrected for non-specific background by subtracting the optical density of the corpus callosum.

### Western blot analysis

The brain tissues were homogenized with lysis buffer (PRO-PREP; iNtRON, Sungnam, Korea; n = 8 mice per group) and centrifuged at 2500 × g for 15 min at 4°C. Equal amounts of total protein (40 mg) isolated from brain tissues were resolved on 8% or 10% sodium dodecyl sulfate polyacrylamide gels and then transferred to nitrocellulose membranes (Hybond ECL; Amersham Pharmacia Biotech, Piscataway, NJ). Membranes were incubated at 4°C for 12 h with the following specific antibodies: anti-GFAP, anti-iba1 (1:1000; Abcam, Inc., Cambridge, MA), anti-COX-2, anti-p38, anti-p-p38, anti-p-JNK, anti-JNK, anti-p-ERK, anti-ERK (Cell Signaling Technology, Inc., Beverly, MA), anti-MAO B (1:1000; Santa Cruz Biotechnologies, Inc., Santa Cruz, CA), and anti-b-actin (1:2500; Santa Cruz Biotechnologies, Inc., Santa Cruz, CA). Blots were then incubated at room temperature for 2 h with corresponding peroxidase-conjugated anti-goat/mouse/rabbit (1/2000; Santa Cruz Biotechnology, Inc., Santa Cruz, CA). Immunoreactive proteins were detected using an enhanced chemiluminescence Western blotting detection system. The relative density of the protein bands was scanned densitometrically using My Image (SLB, Seoul, Korea) and quantified by Lab Works 4.0 (UVP, Upland, CA).

### Electrophoretic mobility shift assay

Gel shift assays were performed according to the manufacturer's recommendations (Promega, Madison, WI). Briefly, 5 × 106 cells were washed twice with 1× PBS, followed by the addition of 1 ml of PBS, and then cells were scraped into a cold Eppendorf tube. Cells were spun down at 13,000 rpm for 5 min, and the resulting supernatant was removed. Cells were suspended in 400 μl of solution A containing 10 mM HEPES, pH 7.9, 1.5 mM MgCl2, 10 mM KCl, 0.5 mM dithiothreitol, 0.2 mM phenylmethylsulfonyl fluoride; vigorously vortexed; allowed to incubate on ice for 10 min; and centrifuged at 12,000 rpm for 6 min. The pelleted nuclei were resuspended in solution C (solution A + 420 mM NaCl, 20% glycerol) and allowed to incubate on ice for 20 min. The cells were centrifuged at 15,000 rpm for 15 min, and the resulting nuclear extract supernatant was collected in a chilled Eppendorf tube. Consensus oligonucleotides were end-labeled using T4 polynucleotide kinase and [γ-32P] ATP for 10 min at 37°C. Gel shift reactions were assembled and allowed to incubate at room temperature for 10 min followed by the addition of 1 μl (50,000-200,000 cpm) of 32P end-labeled oligonucleotide and another 20 min of incubation at room temperature. Subsequently 1 μl of gel loading buffer was added to each reaction and loaded onto a 6% nondenaturing gel and electrophoresis until the dye was four-fifths of the way down the gel. The gel was dried at 80°C for 1 hr and exposed to film overnight at −70°C.

### Statistical analysis

All statistical analysis was performed with GraphPad Prism 4 software (Version 4.03; GraphPad software, Inc., San Diego, CA). Group differences in the escape distance, latency, and velocity in the Morris water maze task were analyzed using *t*-test repeated measures, the factors being treatment and testing day. Otherwise, differences were analyzed by two-way ANOVA followed by Dunnette's *post hoc* test. All values are presented as mean ± SEM. Significance was set at *p* < 0.05 for all tests.

## References

[1] Wu K, Politis M, Piccini P (2009). Parkinson disease and impulse control disorders: a review of clinical features, pathophysiology and management. Postgraduate medical journal.

[2] Okuda K, Kotake Y, Ohta S (2006). Parkinsonism-preventing activity of 1-methyl-1,2,3,4-tetrahydroisoquinoline derivatives in C57BL mouse *in vivo*. Biological & pharmaceutical bulletin.

[3] Langston JW, Ballard P, Tetrud JW, Irwin I (1983). Chronic Parkinsonism in humans due to a product of meperidine-analog synthesis. Science.

[4] Bove J, Zhou C, Jackson-Lewis V, Taylor J, Chu Y, Rideout HJ, Wu DC, Kordower JH, Petrucelli L, Przedborski S (2006). Proteasome inhibition and Parkinson's disease modeling. Annals of neurology.

[5] Dauer W, Przedborski S (2003). Parkinson's disease: mechanisms and models. Neuron.

[6] Exner N, Lutz AK, Haass C, Winklhofer KF (2012). Mitochondrial dysfunction in Parkinson's disease: molecular mechanisms and pathophysiological consequences. The EMBO journal.

[7] Jackson-Lewis V, Przedborski S (2007). Protocol for the MPTP mouse model of Parkinson's disease. Nature protocols.

[8] Boutajangout A, Wisniewski T (2013). The innate immune system in Alzheimer's disease. International journal of cell biology.

[9] Hunter JM, Kwan J, Malek-Ahmadi M, Maarouf CL, Kokjohn TA, Belden C, Sabbagh MN, Beach TG, Roher AE (2012). Morphological and pathological evolution of the brain microcirculation in aging and Alzheimer's disease. PloS one.

[10] Koprich JB, Reske-Nielsen C, Mithal P, Isacson O (2008). Neuroinflammation mediated by IL-1beta increases susceptibility of dopamine neurons to degeneration in an animal model of Parkinson's disease. Journal of neuroinflammation.

[11] Hee Han B, Choi J, Holtzman DM (2002). Evidence that p38 mitogen-activated protein kinase contributes to neonatal hypoxic-ischemic brain injury. Developmental neuroscience.

[12] Krementsov DN, Thornton TM, Teuscher C, Rincon M (2013). The emerging role of p38 mitogen-activated protein kinase in multiple sclerosis and its models. Molecular and cellular biology.

[13] Wong WK, Ou XM, Chen K, Shih JC (2002). Activation of human monoamine oxidase B gene expression by a protein kinase C MAPK signal transduction pathway involves c-Jun and Egr-1. The Journal of biological chemistry.

[14] Li Y, Liu L, Barger SW, Griffin WS (2003). Interleukin-1 mediates pathological effects of microglia on tau phosphorylation and on synaptophysin synthesis in cortical neurons through a p38-MAPK pathway. The Journal of neuroscience.

[15] Baldwin AS (1996). The NF-kappa B and I kappa B proteins: new discoveries and insights. Annual review of immunology.

[16] Barnes PJ, Karin M (1997). Nuclear factor-kappaB: a pivotal transcription factor in chronic inflammatory diseases. The New England journal of medicine.

[17] Haddad JJ (2002). Oxygen-sensitive pro-inflammatory cytokines, apoptosis signaling and redox-responsive transcription factors in development and pathophysiology. Cytokines, cellular & molecular therapy.

[18] Farfara D, Lifshitz V, Frenkel D (2008). Neuroprotective and neurotoxic properties of glial cells in the pathogenesis of Alzheimer's disease. Journal of cellular and molecular medicine.

[19] Fuller S, Steele M, Munch G (2010). Activated astroglia during chronic inflammation in Alzheimer's disease—do they neglect their neurosupportive roles?. Mutation research.

[20] Chiurchiu V, Maccarrone M (2011). Chronic inflammatory disorders and their redox control: from molecular mechanisms to therapeutic opportunities. Antioxidants & redox signaling.

[21] Saha RN, Pahan K (2006). Regulation of inducible nitric oxide synthase gene in glial cells. Antioxidants & redox signaling.

[22] Zarubin T, Han J (2005). Activation and signaling of the p38 MAP kinase pathway. Cell research.

[23] Numagami Y, Sato S, Ohnishi ST (1996). Attenuation of rat ischemic brain damage by aged garlic extracts: a possible protecting mechanism as antioxidants. Neurochemistry international.

[24] Ban JO, Oh JH, Kim TM, Kim DJ, Jeong HS, Han SB, Hong JT (2009). Anti-inflammatory and arthritic effects of thiacremonone, a novel sulfur compound isolated from garlic via inhibition of NF-kappaB. Arthritis research & therapy.

[25] Brunetti L, Menghini L, Orlando G, Recinella L, Leone S, Epifano F, Lazzarin F, Chiavaroli A, Ferrante C, Vacca M (2009). Antioxidant effects of garlic in young and aged rat brain *in vitro*. Journal of medicinal food.

[26] Mahdavi-Roshan M, Zahedmehr A, Mohammad-Zadeh A, Sanati HR, Shakerian F, Firouzi A, Kiani R, Nasrollahzadeh J (2013). Effect of garlic powder tablet on carotid intima-media thickness in patients with coronary artery disease: a preliminary randomized controlled trial. Nutrition and health.

[27] Xiao J, Guo R, Fung ML, Liong EC, Chang RC, Ching YP, Tipoe GL (2013). Garlic-Derived S-Allylmercaptocysteine Ameliorates Nonalcoholic Fatty Liver Disease in a Rat Model through Inhibition of Apoptosis and Enhancing Autophagy. Evidence-based complementary and alternative medicine : eCAM.

[28] Lai YS, Chen WC, Ho CT, Lu KH, Lin SH, Tseng HC, Lin SY, Sheen LY (2014). Garlic essential oil protects against obesity-triggered nonalcoholic fatty liver disease through modulation of lipid metabolism and oxidative stress. Journal of agricultural and food chemistry.

[29] Pintana H, Sripetchwandee J, Supakul L, Apaijai N, Chattipakorn N, Chattipakorn S (2014). Garlic extract attenuates brain mitochondrial dysfunction and cognitive deficit in obese-insulin resistant rats. Applied physiology, nutrition, and metabolism = Physiologie appliquee, nutrition et metabolisme.

[30] Lin GH, Lee YJ, Choi DY, Han SB, Jung JK, Hwang BY, Moon DC, Kim Y, Lee MK, Oh KW, Jeong HS, Leem JY, Shin HK, Lee JH, Hong JT (2012). Anti-amyloidogenic effect of thiacremonone through anti-inflamation *in vitro* and *in vivo* models. Journal of Alzheimer's disease : JAD.

[31] Yun HM, Jin P, Park KR, Hwang J, Jeong HS, Kim EC, Jung JK, Oh KW, Hwang BY, Han SB, Hong JT (2015). Thiacremonone Potentiates Anti-Oxidant Effects to Improve Memory Dysfunction in an APP/PS1 Transgenic Mice Model. Molecular neurobiology.

[32] Ban JO, Hwang CJ, Park MH, Hwang IK, Jeong HS, Lee HP, Hyun BK, Kim JY, Youn HS, Ham YW, Yoon do Y, Han SB, Song MJ, Hong JT (2015). Enhanced cell growth inhibition by thiacremonone in paclitaxel-treated lung cancer cells. Archives of pharmacal research.

[33] Ban JO, Lee DH, Kim EJ, Kang JW, Kim MS, Cho MC, Jeong HS, Kim JW, Yang Y, Hong JT, Yoon do Y (2012). Antiobesity effects of a sulfur compound thiacremonone mediated via down-regulation of serum triglyceride and glucose levels and lipid accumulation in the liver of db/db mice. Phytotherapy research : PTR.

[34] Ban JO, Lee HS, Jeong HS, Song S, Hwang BY, Moon DC, Yoon do Y, Han SB, Hong JT (2009). Thiacremonone augments chemotherapeutic agent-induced growth inhibition in human colon cancer cells through inactivation of nuclear factor-{kappa}B. Molecular cancer research : MCR.

[35] Jo M, Yun HM, Park KR, Park MH, Lee DH, Cho SH, Yoo HS, Lee YM, Jeong HS, Kim Y, Jung JK, Hwang BY, Lee MK, Kim ND, Han SB, Hong JT (2014). Anti-cancer effect of thiacremonone through down regulation of peroxiredoxin 6. PloS one.

[36] Kim EJ, Lee DH, Kim HJ, Lee SJ, Ban JO, Cho MC, Jeong HS, Yang Y, Hong JT, Yoon do Y (2012). Thiacremonone, a sulfur compound isolated from garlic, attenuates lipid accumulation partially mediated via AMPK activation in 3T3-L1 adipocytes. The Journal of nutritional biochemistry.

[37] Roy Choudhury G, Ryou MG, Poteet E, Wen Y, He R, Sun F, Yuan F, Jin K, Yang SH (2014). Involvement of p38 MAPK in reactive astrogliosis induced by ischemic stroke. Brain research.

[38] Pfeiffer RF (2009). Neuroinflammation and Parkinson disease: the silent battleground. Neurology.

[39] Li JY, Ma SS, Huang QY, Li MT (2015). [The Function of Neuroinflammation in Parkinson Disease]. Sheng li ke xue jin zhan [Progress in physiology].

[40] Whitton PS (2007). Inflammation as a causative factor in the aetiology of Parkinson's disease. British journal of pharmacology.

[41] de Pablos RM, Herrera AJ, Espinosa-Oliva AM, Sarmiento M, Munoz MF, Machado A, Venero JL (2014). Chronic stress enhances microglia activation and exacerbates death of nigral dopaminergic neurons under conditions of inflammation. Journal of neuroinflammation.

[42] McGeer PL, Itagaki S, Boyes BE, McGeer EG (1988). Reactive microglia are positive for HLA-DR in the substantia nigra of Parkinson's and Alzheimer's disease brains. Neurology.

[43] Imamura K, Hishikawa N, Sawada M, Nagatsu T, Yoshida M, Hashizume Y (2003). Distribution of major histocompatibility complex class II-positive microglia and cytokine profile of Parkinson's disease brains. Acta neuropathologica.

[44] Shivers KY, Nikolopoulou A, Machlovi SI, Vallabhajosula S, Figueiredo-Pereira ME (2014). PACAP27 prevents Parkinson-like neuronal loss and motor deficits but not microglia activation induced by prostaglandin J2. Biochimica et biophysica acta.

[45] Depboylu C, Stricker S, Ghobril JP, Oertel WH, Priller J, Hoglinger GU (2012). Brain-resident microglia predominate over infiltrating myeloid cells in activation, phagocytosis and interaction with T-lymphocytes in the MPTP mouse model of Parkinson disease. Experimental neurology.

[46] Schiess MC, Barnes JL, Ellmore TM, Poindexter BJ, Dinh K, Bick RJ (2010). CSF from Parkinson disease patients differentially affects cultured microglia and astrocytes. BMC neuroscience.

[47] Wang XJ, Yan ZQ, Lu GQ, Stuart S, Chen SD (2007). Parkinson disease IgG and C5a-induced synergistic dopaminergic neurotoxicity: role of microglia. Neurochemistry international.

[48] Gurler HS, Bilgici B, Akar AK, Tomak L, Bedir A (2014). Increased DNA oxidation (8-OHdG) and protein oxidation (AOPP) by low level electromagnetic field (2. 45 GHz) in rat brain and protective effect of garlic. International journal of radiation biology.

[49] Kumar S (2015). Dual inhibition of acetylcholinesterase and butyrylcholinesterase enzymes by allicin. Indian journal of pharmacology.

[50] Zhu JW, Chen T, Guan J, Liu WB, Liu J (2012). Neuroprotective effects of allicin on spinal cord ischemia-reperfusion injury via improvement of mitochondrial function in rabbits. Neurochemistry international.

[51] Li CY, Cheng XS (2007). [Effects of allicin on changes of hemorheology in focal cerebral ischemia-reperfusion injury]. Zhongguo Zhong yao za zhi = Zhongguo zhongyao zazhi = China journal of Chinese materia medica.

[52] Batirel HF, Naka Y, Kayano K, Okada K, Vural K, Pinsky DJ, Oz MC (2002). Intravenous allicin improves pulmonary blood flow after ischemia-reperfusion injury in rats. The Journal of cardiovascular surgery.

[53] Chen YQ, Pan WH, Liu JH, Chen MM, Liu CM, Yeh MY, Tsai SK, Young MS, Zhang XM, Chao HM (2012). The effects and underlying mechanisms of S-allyl l-cysteine treatment of the retina after ischemia/reperfusion. Journal of ocular pharmacology and therapeutics.

[54] Ashafaq M, Khan MM, Shadab Raza S, Ahmad A, Khuwaja G, Javed H, Khan A, Islam F, Siddiqui MS, Safhi MM, Islam F (2012). S-allyl cysteine mitigates oxidative damage and improves neurologic deficit in a rat model of focal cerebral ischemia. Nutrition research.

[55] Kim YR, Lee NJ, Ban JO, Yoo HS, Lee YM, Yoon YP, Eum SY, Jeong HS, Yoon DY, Han SB, Hong JT (2013). Curative Effects of Thiacremonone against Acetaminophen-Induced Acute Hepatic Failure via Inhibition of Proinflammatory Cytokines Production and Infiltration of Cytotoxic Immune Cells and Kupffer Cells. Evidence-based complementary and alternative medicine : eCAM.

[56] Ehringer H, Hornykiewicz O (1960). [Distribution of noradrenaline and dopamine (3-hydroxytyramine) in the human brain and their behavior in diseases of the extrapyramidal system]. Klinische Wochenschrift.

[57] Silver D (2006). Impact of functional age on the use of dopamine agonists in patients with Parkinson disease. The neurologist.

[58] Ogasahara S, Nishikawa Y, Takahashi M, Wada K, Nakamura Y, Yorifuji S, Tarui S (1984). Dopamine metabolism in the central nervous system after discontinuation of L-dopa therapy in patients with Parkinson disease. Journal of the neurological sciences.

[59] Jenner WN, Rose FA (1974). Dopamine 3-O-sulphate, an end product of L-dopa metabolism in Parkinson patients. Nature.

[60] Kastner A, Hirsch EC, Agid Y, Javoy-Agid F (1993). Tyrosine hydroxylase protein and messenger RNA in the dopaminergic nigral neurons of patients with Parkinson's disease. Brain research.

[61] Riederer P, Konradi C, Youdim MB (1990). The role of MAO in dopaminergic transmission. Advances in neurology.

[62] Rojas P, Serrano-Garcia N, Medina-Campos ON, Pedraza-Chaverri J, Maldonado PD, Ruiz-Sanchez E (2011). S-Allylcysteine, a garlic compound, protects against oxidative stress in 1-methyl-4-phenylpyridinium-induced parkinsonism in mice. The Journal of nutritional biochemistry.

[63] Karunakaran S, Saeed U, Mishra M, Valli RK, Joshi SD, Meka DP, Seth P, Ravindranath V (2008). Selective activation of p38 mitogen-activated protein kinase in dopaminergic neurons of substantia nigra leads to nuclear translocation of p53 in 1-methyl-4-phenyl-1,2,3,6-tetrahydropyridine-treated mice. The Journal of neuroscience.

[64] Ferrer I, Blanco R, Carmona M, Puig B, Barrachina M, Gomez C, Ambrosio S (2001). Active, phosphorylation-dependent mitogen-activated protein kinase (MAPK/ERK), stress-activated protein kinase/c-Jun N-terminal kinase (SAPK/JNK), and p38 kinase expression in Parkinson's disease and Dementia with Lewy bodies. Journal of neural transmission.

[65] Williams JA, Pontzer CH, Shacter E (2000). Regulation of macrophage interleukin-6 (IL-6) and IL-10 expression by prostaglandin E2: the role of p38 mitogen-activated protein kinase. Journal of interferon & cytokine research.

[66] Baldassare JJ, Bi Y, Bellone CJ (1999). The role of p38 mitogen-activated protein kinase in IL-1 beta transcription. Journal of immunology.

[67] Foey AD, Parry SL, Williams LM, Feldmann M, Foxwell BM, Brennan FM (1998). Regulation of monocyte IL-10 synthesis by endogenous IL-1 and TNF-alpha: role of the p38 and p42/44 mitogen-activated protein kinases. Journal of immunology.

[68] Lahti A, Kankaanranta H, Moilanen E (2002). P38 mitogen-activated protein kinase inhibitor SB203580 has a bi-directional effect on iNOS expression and NO production. European journal of pharmacology.

[69] Chen C, Chen YH, Lin WW (1999). Involvement of p38 mitogen-activated protein kinase in lipopolysaccharide-induced iNOS and COX-2 expression in J774 macrophages. Immunology.

[70] De Zutter GS, Davis RJ (2001). Pro-apoptotic gene expression mediated by the p38 mitogen-activated protein kinase signal transduction pathway. Proceedings of the National Academy of Sciences of the United States of America.

[71] Kyriakis JM (1999). Activation of the AP-1 transcription factor by inflammatory cytokines of the TNF family. Gene expression.

[72] Jha SK, Jha NK, Kar R, Ambasta RK, Kumar P (2015). p38 MAPK and PI3K/AKT Signalling Cascades inParkinson's Disease. International journal of molecular and cellular medicine.

[73] Xing B, Liu M, Bing G (2007). Neuroprotection with pioglitazone against LPS insult on dopaminergic neurons may be associated with its inhibition of NF-kappaB and JNK activation and suppression of COX-2 activity. Journal of neuroimmunology.

[74] Saporito MS, Thomas BA, Scott RW (2000). MPTP activates c-Jun NH(2)-terminal kinase (JNK) and its upstream regulatory kinase MKK4 in nigrostriatal neurons *in vivo*. Journal of neurochemistry.

[75] Lawrence T (2009). The nuclear factor NF-kappaB pathway in inflammation. Cold Spring Harbor perspectives in biology.

[76] Olson CM, Hedrick MN, Izadi H, Bates TC, Olivera ER, Anguita J (2007). p38 mitogen-activated protein kinase controls NF-kappaB transcriptional activation and tumor necrosis factor alpha production through RelA phosphorylation mediated by mitogen- and stress-activated protein kinase 1 in response to Borrelia burgdorferi antigens. Infection and immunity.

[77] Choi DY, Lee MK, Hong JT (2012). Lack of CCR5 modifies glial phenotypes and population of the nigral dopaminergic neurons, but not MPTP-induced dopaminergic neurodegeneration. Neurobiology of disease.

